# Successful conservative management of symptomatic bilateral dorsal patellar defects presenting with cartilage involvement and bone marrow edema: MRI findings

**DOI:** 10.1007/s00256-016-2335-5

**Published:** 2016-01-26

**Authors:** Thomas C. Kwee, Heleen Sonneveld, Maarten Nix

**Affiliations:** Department of Radiology and Nuclear Medicine, University Medical Center Utrecht, Heidelberglaan 100, 3584 CX Utrecht, The Netherlands; Department of Orthopaedics, Meander Medical Center, Amersfoort, The Netherlands; Department of Radiology, Meander Medical Center, Amersfoort, The Netherlands

**Keywords:** Bilateral, Cartilage, Dorsal patellar defect, Edema, MRI, X-ray

## Abstract

The dorsal patellar defect is a relatively rare entity that involves the superolateral quadrant of the patella. It is usually considered to represent a delayed ossification process, although its exact origin remains unclear. Because of its usually innocuous nature and clinical course, invasive interventions are generally deemed unnecessary, although curretage has been successfully performed on symptomatic cases. This case report presents a rather unusual case of symptomatic bilateral dorsal patellar defects with cartilage involvement and widespread surrounding bone marrow edema as demonstrated by magnetic resonance imaging (MRI). Both cartilage involvement and bone marrow edema should be considered part of the spectrum of associated MRI findings that can be encountered in this entity. Furthermore, the presented case shows that symptomatic dorsal patellar defects can be treated conservatively with success and that (decrease of) pain symptoms are likely related to (decrease of) bone marrow edema.

## Introduction

The patella is the largest sesamoid bone of the skeleton and is formed and located in between the quadriceps femoris and patellar tendons [1]. Its principal role is to facilitate and optimize the extensor function of the quadriceps muscle. Furthermore, it protects the ventral cartilage surfaces of the knee joint [1]. The patella initially ossifies at between 3 and 5 years, commencing as multiple foci that rapidly coalesce [2]. As the patellar ossification center enlarges, the expanding margins may be irregular and associated with accessory ossification centers [2]. These are most commonly located superolaterally and may lead to the development of a multipartite patella, with bipartite patella being the most common variation [2]. The multipartite patella has cartilaginous continuity despite the appearance of osseous discontinuity [2]. Another anomaly of the patella, thought to be closely related to the multipartite patella, is the so-called dorsal patellar defect. Characteristically, the dorsal patellar defect consists of a lytic and round lesion with well-defined margins located subchondrally in the superolateral quadrant of the patella [3]. The majority of dorsal patellar defects are discovered incidentally, asymptomatic, and of no clinical significance [3]. At magnetic resonance imaging (MRI) and arthrography, the overlying articular cartilage is described to appear intact [4, 5]. However, this is not always the case [6–9]. Meanwhile, to the best of our knowledge, the occurrence of bone marrow edema surrounding the dorsal patellar defect has not yet been described. We report a case of a teenager who presented with pain in both knees and had bilateral dorsal patellar defects with overlying cartilage involvement and surrounding bone marrow edema at MRI. After 8 months of conservative management, he was almost symptom-free and repeated MRI showed markedly decreased bone marrow edema and progressive “filling” of the dorsal defects of the patella.

## Case report

**Fig. 1:**
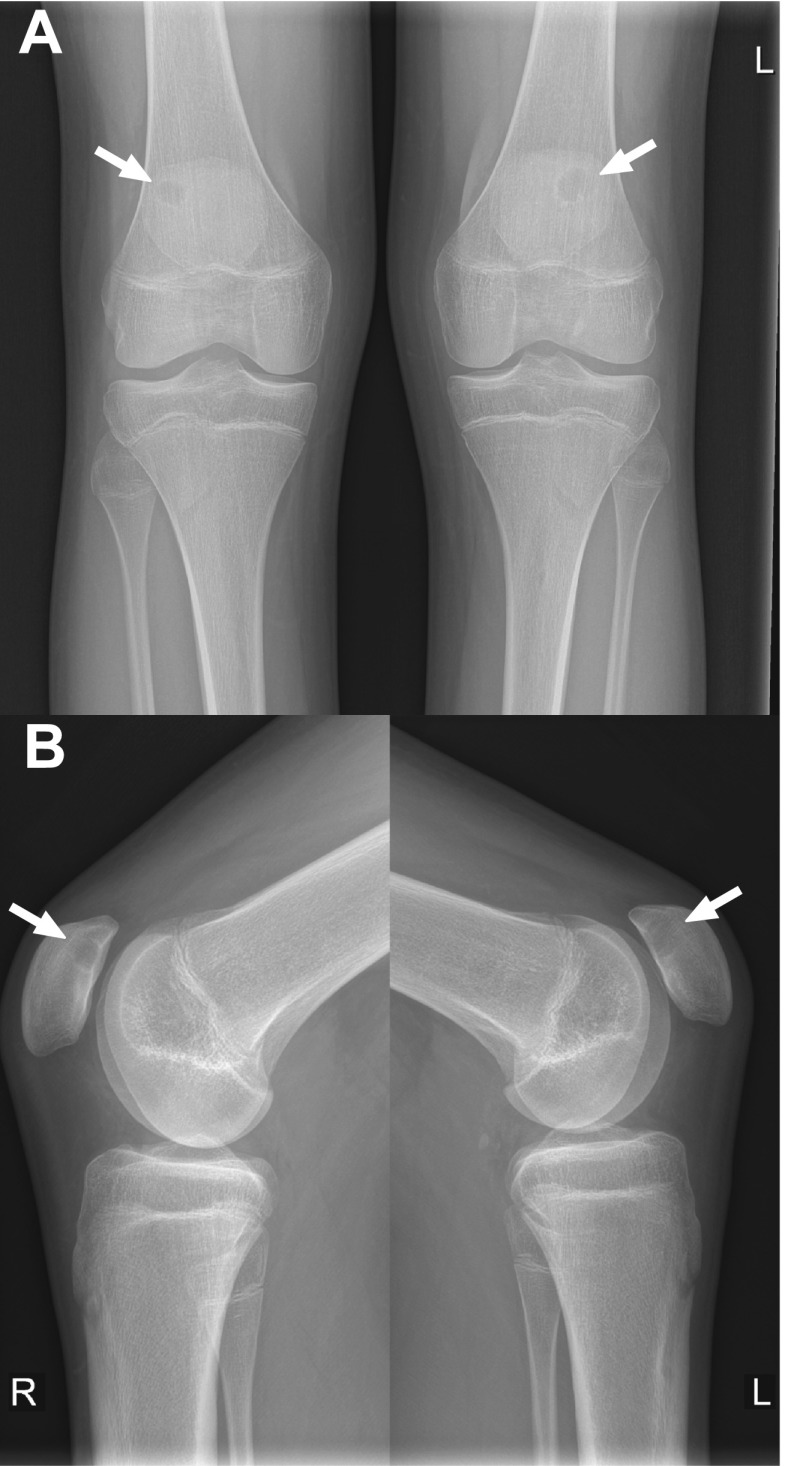
Frontal (**a**) and lateral (**b**) radiographs of both knees demonstrate a lytic and round lesion with well-defined margins in the subchondral regions of the superolateral quadrant of both patellae (arrows)

**Fig. 2:**
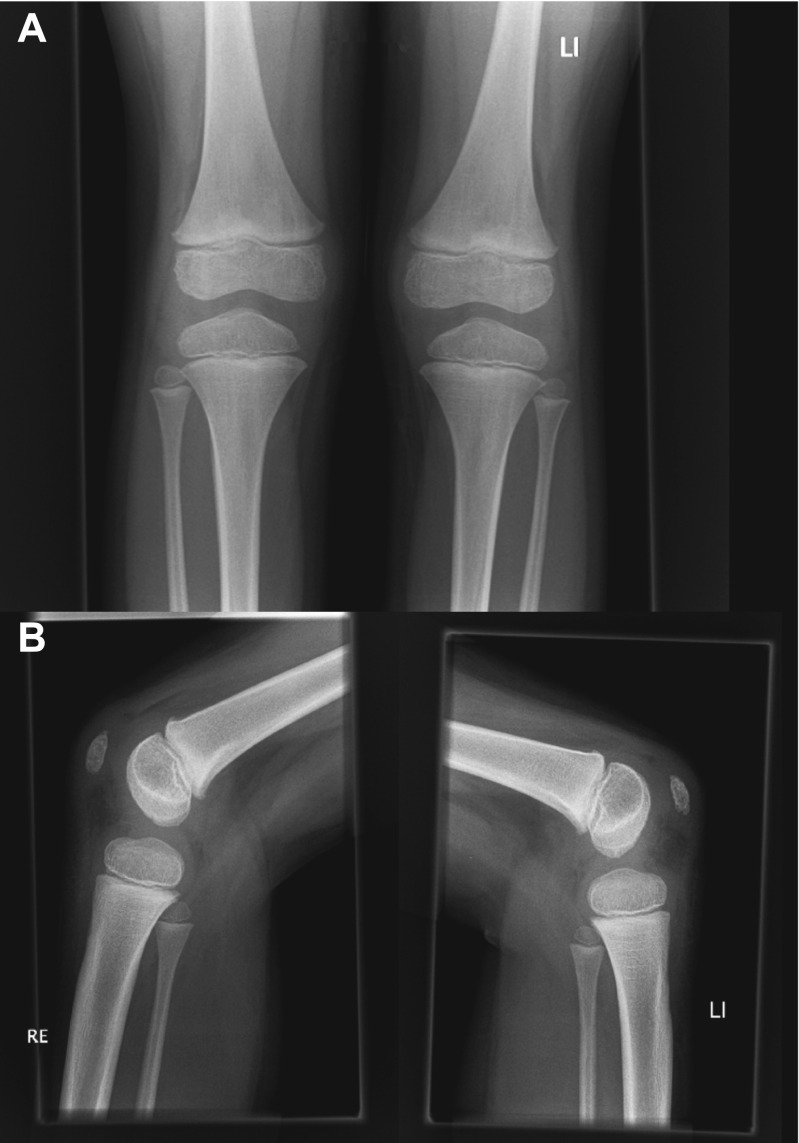
Frontal (**a**) and lateral (**b**) radiographs of both knees that were made 9.5 years earlier show no abnormalities

**Fig. 3:**
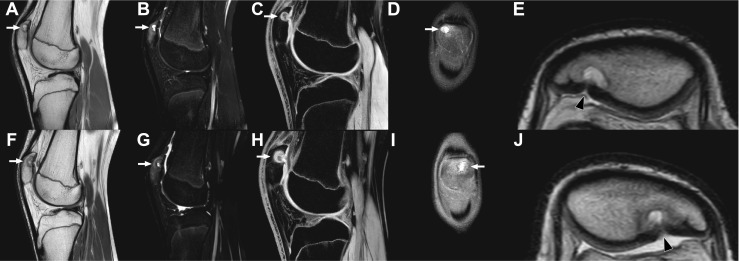
MRI of the right knee (**a**–**e**) and left knee (**f**–**j**) with sagittal proton-density (**a**, **f**), sagittal T2-weighted spectral attenuated inversion recovery (**b**, **g**), sagittal 3D water selective fluid (**c**, **h**), coronal proton-density (**d**, **i**), and magnified axial proton-density (**e**, **j**) slices shown. The lesion in the superolateral quadrant of both patellae, consistent with dorsal patellar defect, is demonstrated (arrows). Note the associated cartilage involvement with a slit-like defect/apparent discontuinity on both retropatellar surfaces (**e**, **j**, arrowheads). Also note the widespread surrounding high T2 signal in both patellae, consistent with bone marrow edema (**b**, **g**). MRI did not show any other abnormalities in either knee

**Fig. 4:**
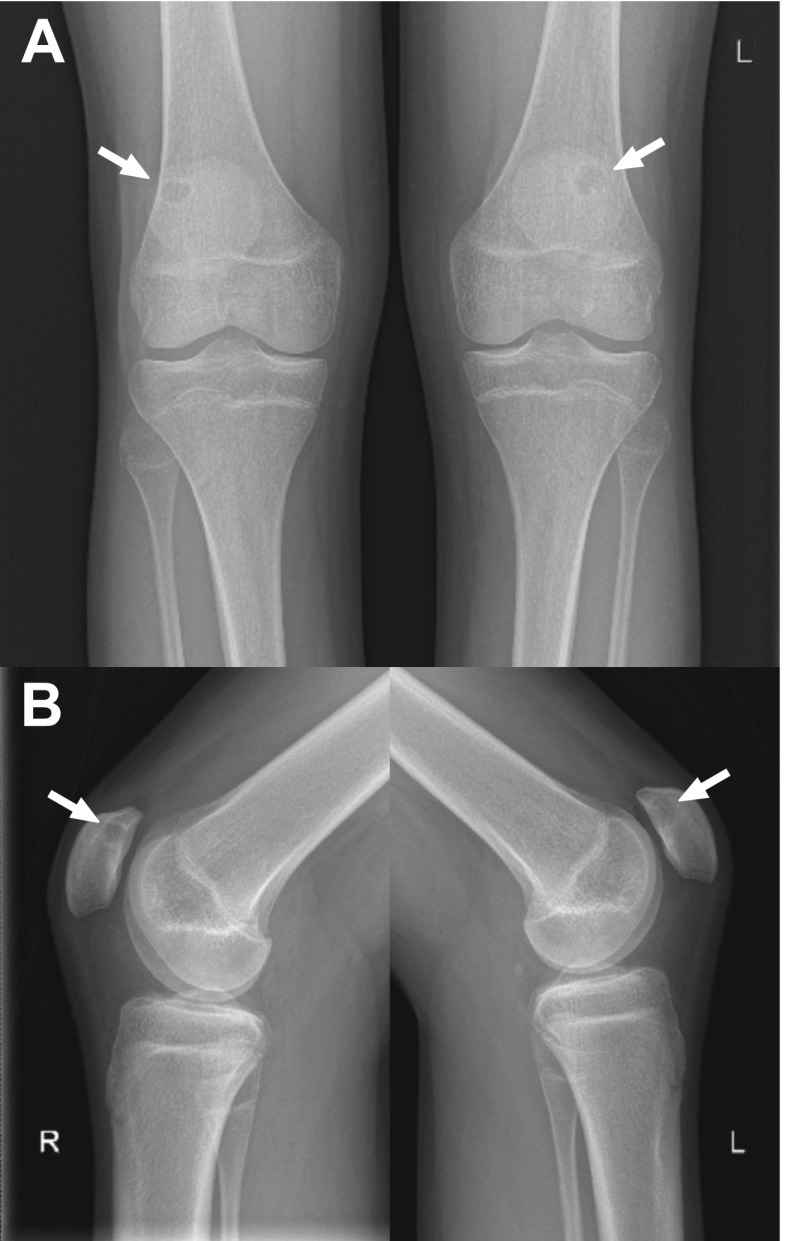
Eight-month follow-up frontal (**a**) and lateral (**b**) radiographs of both knees still demonstrate the dorsal patellar defects (arrows), without any obvious changes compared to 8 months earlier (Fig. 1). However, the patient was almost symptom-free at the time of this radiographic examination

**Fig. 5:**
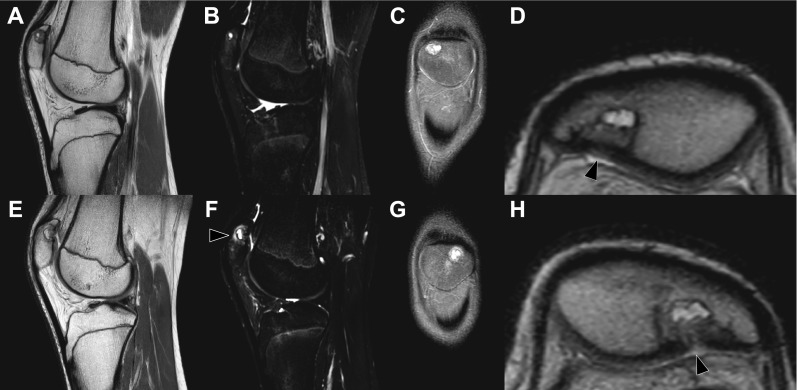
Eight-month follow-up MRI of the right knee (**a**–**d**) and left knee (**e**–**h**) with sagittal proton-density (**a**, **e**), sagittal T2-weighted spectral attenuated inversion recovery (**b**, **f**), oronal proton-density slices (**c**, **g**), and magnified axial proton-density views of the patellae (**d**, **h**) shown. The dorsal patellar defects are still present, but the surrounding bone marrow edema has decreased considerably (**b**, **f**) compared to 8 months earlier (Fig. 3). Only in the left knee some noteworthy bone marrow edema is still seen (**f**, arrowhead). Also note progressive “filling” of the dorsal defects with apparent (onset of) “closure” of the slit-like defects/discontuinities on both retropatellar surfaces (**d**, **h**, arrowheads) compared to 8 months earlier (Fig. 3). The patient was almost symptom-free at the time of this MRI examination

A 16-year-old otherwise healthy male (height: 189.5 cm, weight: 68 kg) was referred to the orthopaedic department because of bilateral knee pain and clunking. These symptoms occurred during running and other forceful movements, were less but not absent with rest, and had been present for more than 1 year. There was no history of trauma. Physiotherapy was reported to be not effective. Other than pain on palpation of the inferior pole of the patella on both sides, the physical examination was unremarkable (i.e., normal gait pattern, normal range of motion of both knees, no signs of hydrops, no ligamentous laxity, no signs of meniscal injury). Radiographs of both knees showed a lytic and round lesion with well-defined margins in the subchondral regions of the superolateral quadrant of both patellae, in keeping with bilateral dorsal patellar defects (Fig. 1). Interestingly, radiographs of both knees that were made because of chronic knee pain 9.5 years earlier, at the age of 6.5 years, showed no abnormalities (Fig. 2). MRI of both knees was performed for further evaluation and to exclude other causes of knee symptoms, using a 1.5-T system (Ingenia, Philips Healthcare) with a 16-channel knee coil. MRI not only demonstrated the bilateral dorsal patellar defects, but also showed an associated deep slit-like cartilage defect with widespread surrounding bone marrow edema in both patellae (Fig. 3). Note that MRI did not show any other causes for the symptoms than the bilateral patellar defects with extensive surrounding bone marrow edema and apparent retropatellar cartilage discontinuties. Because of the usual innocuous nature and clinical course of the dorsal patellar defect, conservative management was chosen (i.e., physiotherapy and instruction to avoid heavy exercise) rather than further invasive (diagnostic or therapeutic) interventions, and the patient was referred back to his general practitioner. The patient returned for a follow-up clinical consultation, radiographic and MRI examinations after 8 months. Radiographs of both knees still showed the dorsal patellar defects, without any obvious changes compared to 8 months earlier (Fig. 4). However, MRI of both knees showed a considerable decrease in the amount of surrounding bone marrow edema compared to 8 months earlier (Fig. 5). In addition, MRI showed progressive “filling” of the dorsal patellar defects with apparent (onset of) “closure” of the slit-like defects/discontuinities on both retropatellar surfaces compared to 8 months earlier (Fig. 5). Importantly, the patient was almost symptom-free. Therefore, the conservative management could be considered successful.

## Discussion

The dorsal patellar defect was first reported by Caffey and Keats in the early 1970s [10, 11]. A study by Johnson and Brogdon [12] aimed to determine the incidence of this phenomenon by reviewing the radiographs of 2,349 knees in 1,192 consecutive (both asymptomatic and symptomatic) patients. They observed 13 dorsal patellar defects in 12 individuals, with ages ranging between 17 and 56 years [12]. When both knees were available for evaluation, the lesion was bilateral in only one of three affected individuals [12]. Johnson and Brogdon [12] concluded this entity to be present in about 1 % of the population and that it may be found at any age ranging from the preadolescent to mature adult. In yet another study by Van Holsbeeck et al. [6], 6 dorsal patellar defects were identified in a series of 2,286 single radiographic examinations of the knee made because of different complaints, yielding an incidence of 0.26 %.

Despite the fact that the phenomenon of the dorsal patellar defect has been known for over 40 years, its origin still remains unclear. Different hypotheses have been postulated. Since the dorsal patellar defect is located in the superolateral quadrant of the patella, similarly to the multipartite patella, and previous studies have reported cases in which both entities coexist [6, 7], it is generally considered to represent a developmental anomaly of the epiphysis with delayed ossification. Van Holsbeeck et al. [6] hypothesized stress phenomena in the region of the insertion of the vastus lateralis muscle and accompanying vascular insufficiency in the superolateral quadrant of the patella to be responsible for delayed ossification. In another report by Sugita et al. [13], who excised the entire lesion together with covering articular cartilage in three cases with a dorsal patellar defect, it was speculated that this lesion originates from ischemic episodes followed by subchondral collapse and consequent active reparative reaction. Despite its unclear (probably complex and multifactorial) etiology, the dorsal patellar defect does not grow in size and usually heals spontaneously with sclerosis, thus requiring no further intervention [3]. However, curettage has been performed on symptomatic cases with success [9, 14, 15].

The diagnosis of dorsal patellar defect is generally straightforward. Because of its typical imaging features and location, it can usually be differentiated from pathologic conditions such as giant cell tumor, chondroblastoma, aneurysmal bone cyst, osteomyelitis, osteoid osteoma, solitary bone cyst, intraosseous gout, metastasis, intraosseous ganglion, and brown tumor in hyperparathyroidism [16]. In addition, the lesion should not be mistaken for osteochondritis dissecans, which has a predilection for the medial facet of the patella and may have a separated articular cartilage flap [17]. Unlike its characteristics at radiography, however, the associated MRI findings of the dorsal patellar defect have been less well described. Although both initial [4] and more recent reports [5] describe the overlying cartilage to be intact, this is not always the case, as illustrated by the presented patient (despite the lack of arthroscopic confirmation) and also by some previous reports [6–9]. Van Holsbeeck et al. [6] consider the association of the dorsal patellar defect and cartilaginous defect rare, with the latter being an epiphenomenon of the former, and claim that it is only found in symptomatic patients. Interestingly, in the presented case with bilateral dorsal patellar defects, extensive surrounding bone marrow edema was also observed. To the best of our knowledge, this MRI feature has not been previously described. The follow-up clinical and MRI examinations after 8 months provided important additional information because they demonstrate that symptomatic dorsal patellar defects can be treated conservatively and that (decrease of) pain symptoms are very likely related to (decrease of) bone marrow edema. Note that edematous changes in other sesamoids and accessory bones, as demonstrated by MRI, have also been reported to be associated with pain symptoms [18–20]. The cause of the surrounding patellar bone marrow edema and whether it is primary or secondary to the dorsal patellar defect are still unclear, however.

In conclusion, this report described a rather unusual case of bilateral dorsal patellar defects with cartilage involvement and extensive surrounding bone marrow edema as demonstrated by MRI. The latter two should be considered as part of the spectrum of associated MRI findings that can be encountered in this entity. Furthermore, the presented case shows that symptomatic dorsal patellar defects can be treated conservatively with success and that (decrease of) pain symptoms are likely related to (decrease of) bone marrow edema.
